# Sex Ratio at Birth in Northern Ireland During the COVID‐19 Pandemic: A Comparison With Published Data From the Republic of Ireland, England and Wales

**DOI:** 10.1002/ajhb.70099

**Published:** 2025-06-27

**Authors:** Gwinyai Masukume, Peyton Cleaver, Roy K. Philip, Victor Grech, Amy L. Non

**Affiliations:** ^1^ School of Public Health, Physiotherapy and Sports Science University College Dublin Dublin Ireland; ^2^ Department of Anthropology University of California San Diego La Jolla California USA; ^3^ Division of Neonatology, Department of Paediatrics University Maternity Hospital Limerick Limerick Ireland; ^4^ University of Limerick School of Medicine Limerick Ireland; ^5^ Academic Department of Paediatrics Medical School, Mater Dei Hospital Msida Malta

**Keywords:** COVID‐19 pandemic, male births, Northern Ireland, sex ratio, stress

## Abstract

**Objectives:**

The COVID‐19 pandemic has been linked in several countries to fluctuations in the proportion of male live births/total live births, known as the sex ratio at birth (SRB). This study investigates how the pandemic influenced SRB patterns in Northern Ireland compared to published data from neighboring regions, including the Republic of Ireland with which it shares an open land border, and England and Wales, across the sea.

**Methods:**

Monthly live birth data for Northern Ireland from 2015 to 2021 were obtained from the Northern Ireland Statistics and Research Agency. A time series analysis predicted the SRB for 2020 using data from 2015 to 2019. Predicted and observed SRB values were compared for 2020.

**Results:**

In August 2020, 5 months after the pandemic declaration, the SRB fell significantly to 49.13%, the period's lowest, below the 95% prediction interval (50.09%–51.85%). In December 2020, 9 months after the declaration, the SRB rose to 54.48%, exceeding the prediction interval (49.75%–51.57%). This overall SRB pattern resembled that in England and Wales but differed from the Republic of Ireland.

**Conclusion:**

The decline in SRB in August 2020, occurring 3–5 months after the pandemic declaration, suggests the pandemic disproportionately affected male fetuses in Northern Ireland. The rise in December, 9 months after the declaration, may relate to increased sexual activity in March 2020 following lockdown in a subset of the population. Northern Ireland's SRB pattern aligns more with England and Wales than the Republic of Ireland, indicating that socio‐political ties in the United Kingdom may be more influential for pandemic response than geographical proximity.

## 1 Introduction

The sex ratio at birth (SRB), also known as the secondary sex ratio, is defined as the proportion of live male births to total live births and serves as a vital demographic measure as well as an indicator of long‐term health outcomes (Davis et al. 1998; Nilsson et al. 2024). Typically, in humans, live male births slightly outnumber live female births (Chao et al. 2019). However, the SRB can experience fluctuations in response to significant external stressors, including natural disasters, terrorist attacks, economic instability, and health crises (Fontanesi et al. 2024; Masukume et al. 2017; Mikulec et al. 2023). The COVID‐19 pandemic, declared by the World Health Organization on March 11, 2020 (Cucinotta and Vanelli 2020), stands out as an unprecedented disruption, prompting extensive research into SRB variations across diverse regions, including Africa (Masukume et al. 2022), Asia (Inoue and Mizoue 2022; Saadat 2021), the Americas (Bruckner et al. 2023; Cleaver and Non 2024; Moreno et al. 2024), and Europe (Masukume et al. 2023; Masukume et al. 2024; Pavić 2024).

Northern Ireland provides a unique context for examining these SRB fluctuations. While sharing the island of Ireland with the Republic of Ireland, Northern Ireland is politically part of the United Kingdom, alongside England and Wales, which are located across the Irish Sea (Royle 2012). Despite the lack of a physical border between Northern Ireland and the Republic of Ireland, there are complex socio‐political distinctions which add a layer of complexity to their demographic dynamics (Ahmed and May 2021). This socio‐political distinction, coupled with differing public health responses to the pandemic, establishes Northern Ireland—and the island of Ireland as a whole—as a valuable natural experiment for comparing SRB trends both within the island and with those in neighboring regions (Grech 2015).

In both Northern Ireland and the Republic of Ireland, initial confirmed cases of COVID‐19 were reported in the final week of February 2020, with the first recorded deaths following in mid‐March (Kennelly et al. 2020; Northern Ireland Statistics and Research Agency 2023; Perumal et al. 2020). By March, as the pandemic's impact deepened, heightened levels of anxiety and depression became prevalent across both populations (Hyland et al. 2020; McPherson et al. 2021).

There were notable similarities in COVID‐19 restrictions between the Republic of Ireland and Northern Ireland. For instance, both jurisdictions announced the cancellation of St Patrick's Day parades on March 9, 2020, and by 24 March, both prohibited all sporting events as well as indoor and outdoor gatherings of any size (Nolan et al. 2021). However, there were also key differences, as Northern Ireland's government prioritized coordination with the United Kingdom over alignment with the Republic of Ireland's measures (Ahmed and May 2021). For instance, while schools and higher education institutions in the Republic of Ireland closed on March 12, 2020, Northern Ireland, more closely aligned with the United Kingdom than the Republic, followed with similar closures nearly a week later (Nolan et al. 2021). March 2020, thus, became a pivotal month, marked by the first fatalities, widespread psychological impact, and the implementation of major public health measures across the island.

Drawing on our previously published data, in England and Wales, the SRB declined in June 2020, 3 months after the pandemic declaration, followed by an increase in December 2020 (Masukume et al. 2023). In contrast, the Republic of Ireland showed a different pattern, with the SRB falling in December 2020, 9 months after the declaration (Masukume et al. 2024).

In exploring the fluctuations of the SRB, the Trivers‐Willard hypothesis offers potentially valuable biological insight. This hypothesis proposes that male fetuses are more vulnerable to unfavorable conditions, affecting their survival and, consequently, the observed SRB (Trivers and Willard 1973). Under stressful or unfavorable circumstances, this hypothesis suggests that female offspring may be favored due to their higher survival potential, and greater likelihood to successfully reproduce, leading to a decline in male births. Conversely, in more favorable environments, the likelihood of male births may increase, as males typically enjoy greater reproductive success. The differing aforementioned pandemic response patterns observed between the Republic of Ireland and England and Wales highlight the importance of examining how the pandemic may have impacted the SRB in Northern Ireland. Analyzing the SRB in Northern Ireland not only reveals the local demographic effects of the pandemic but also offers insights into how socio‐political and geographical factors influence reproductive biology.

## 2 Methods

This study analyzed monthly live birth data for Northern Ireland, obtained from the Northern Ireland Statistics and Research Agency (NISRA), covering all recorded live births from January 1, 2015, to 31 December 2021. The data are publicly available and anonymized, negating the need for ethical approval. The time period and methodological approach align with those in our previous studies on the Republic of Ireland and England and Wales, ensuring comparability across regions (Masukume et al. 2023; Masukume et al. 2024).

Given the relatively small number of births in Northern Ireland, the SRB demonstrates greater month‐to‐month variability, with values occasionally falling outside typical reference ranges (Chao et al. 2019). Such variability is a known feature of analyses based on smaller populations, where statistical estimates are inherently less stable (Button et al. 2013). To address this, we incorporated domain‐specific knowledge to guide model selection and interpretation, favoring an approach that remained consistent with established SRB reference ranges and best practices for time series analysis in low‐power settings (Lai and Wong 2006).

To predict the SRB for 2020, we applied a time series decomposition model, using 2015–2019 as a baseline. This 5‐year period (60 months) reflects a common duration used in COVID‐19 forecasting studies (Achilleos et al. 2022). We focused our analysis on estimating the SRB for each month of 2020. The model followed a multiplicative structure (*Y*=*S* × *T* × *I*), where *Y* represents the SRB, with components *S*, *T*, and *I* representing seasonal patterns, long‐term trends, and irregular variations, respectively (Hyndman and Athanasopoulos 2018). This decomposition isolated the contributors to the SRB pattern, enhancing the robustness of SRB predictions for 2020.

We first checked for stationarity using the Dickey–Fuller test, which confirmed stationarity (*p* < 0.001). Based on the multiplicative model, we calculated 95% prediction intervals for SRB values in each month of 2020.

We also applied an autoregressive moving average (ARMA) model with autoregressive and moving average parameters of [5] each, to predict SRBs for 2020, and selected these model parameters based on the lowest Akaike Information Criterion (AIC) value (Table S1). The 95% prediction intervals were calculated using Stata's “predict” command with the “mse” option. The ARMA model was less effective in capturing real‐world SRB reference ranges (Chao et al. 2019), with its lower bound often below 50%, unlike the multiplicative time series model, which consistently predicted bounds above this threshold. While ARMA models generally perform best with typically over 100 monthly observations (Box and Tiao 1975), performance can decline when the historical data span is temporally distant from the forecast period. Statistical analysis was performed using Stata version 17BE (College Station, TX, USA).

## 3 Results

**FIGURE 1:**
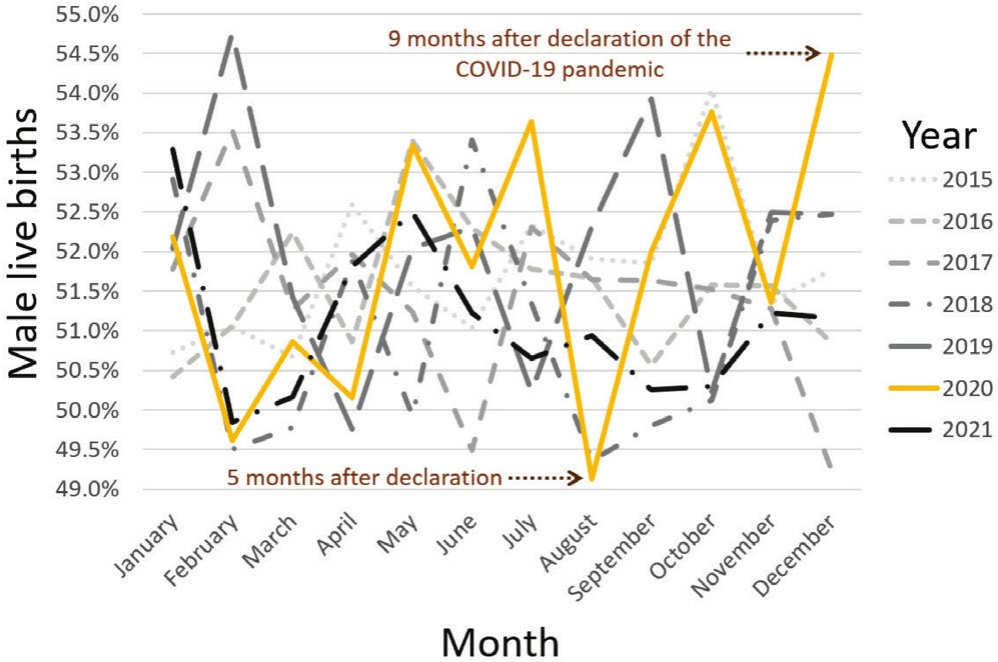
Monthly proportions of male live births from January 2015 to December 2021. The solid orange line highlights data for 2020, while the lines for other years are differentiated as shown in the key.

From January 2015 to December 2021, a total of 160 081 live births were recorded, comprising 82 497 male and 77 584 female births, with an overall SRB of 51.53%. The lowest recorded monthly SRB in the study period occurred in August 2020, with a ratio of 49.13% (96.56 males per 100 females) (Figure 1), which was below the 95% prediction interval of 50.09%–51.85% (Table 1).

**TABLE 1:** Observed and predicted sex ratio at birth for each month of 2020.

Month	Male live births	Female live births	Observed SRB	Predicted SRB	Lower 95% PI	Upper 95% PI
January	1005	921	52.18%	51.47%	50.64%	52.30%
February	827	840	49.61%	51.91%	51.06%	52.75%
March	884	854	50.86%	50.91%	50.07%	51.74%
April	829	824	50.18%	50.83%	49.99%	51.68%
May	941	823	53.34%	52.56%	51.67%	53.44%
June	900	837	51.81%	51.61%	50.74%	52.49%
July	1008	871	53.65%	51.13%	50.25%	52.00%
August	899	931	49.13%	50.97%	50.09%	51.85%
September	1003	926	52.00%	51.23%	50.34%	52.12%
October	1037	892	53.76%	50.61%	49.72%	51.50%
November	892	845	51.35%	51.62%	50.71%	52.54%
December	906	757	54.48%	50.66%	49.75%	51.57%

Abbreviations: PI: prediction interval; SRB: sex ratio at birth.

**FIGURE 2:**
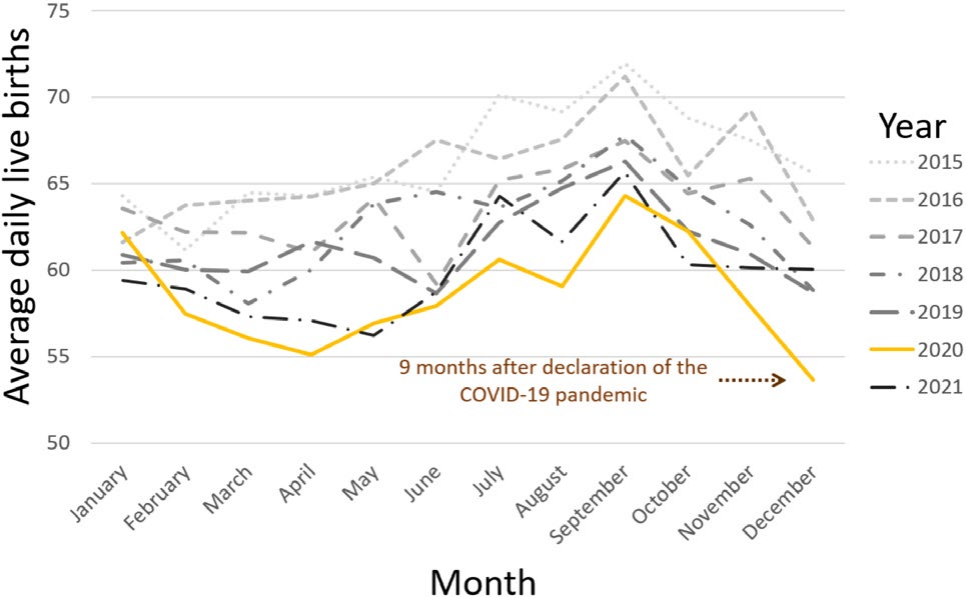
Daily average total live births from January 2015 to December 2021. The solid orange line represents data for 2020, while the lines for other years are shown as indicated in the key.

**FIGURE 3:**
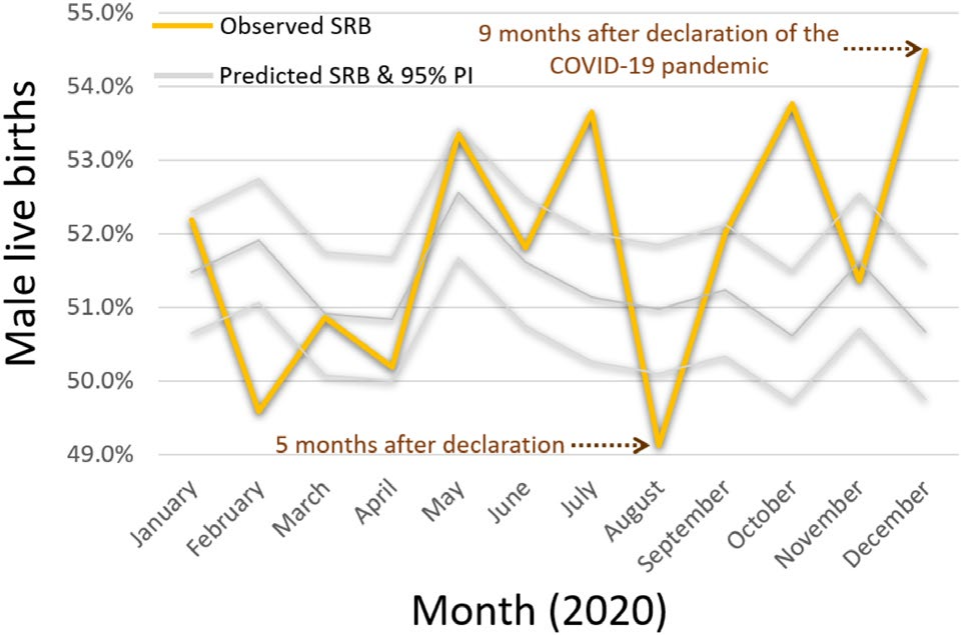
Observed and predicted sex ratio at birth (SRB) for 2020, with the 95% prediction interval (PI) indicated.

December 2020 showed the lowest average daily births across the study period, with an average of 54 births per day (Figure 2). December 2020 also recorded the highest SRB for any December in the study period at 54.48% (119.68 males per 100 females), and was the peak SRB for 2020. This SRB value was above the 95% prediction interval, which was 49.75%–51.57% (Table 1 and Figure 3).

July and October 2020 recorded SRBs of 53.65% (115.73 males per 100 females) and 53.76% (116.26 males per 100 females), respectively, both above their 95% prediction intervals of 50.25%–52.00% and 49.72%–51.50% (Table 1 and Figure 3).

## 4 Discussion

This study finds that the declaration of the COVID‐19 pandemic in March 2020 was associated with notable shifts in the SRB in Northern Ireland. Specifically, August 2020 exhibited the lowest monthly SRB recorded in the study period, at 49.13% (96.56 males per 100 females), a significant decline that aligned with the expected 3‐ to 5‐month lag commonly associated with SRB reductions after major stressful population events (Masukume et al. 2017; Retnakaran and Ye 2020). This drop in SRB during August 2020 likely reflects increased maternal stress during the second trimester of these pregnancies, which had a disproportionate effect on male fetuses, leading to a higher male fetal loss rate (Bruckner et al. 2010). In England and Wales, there was a similar decline in the SRB in June 2020, 3–5 months after the pandemic's onset, mirroring the trend observed in Northern Ireland (Masukume et al. 2023). Contrastingly, the Republic of Ireland saw no such drop in the SRB 3–5 months following the onset of the pandemic, raising questions about the varying effects of the pandemic and public health measures on population stress in different contexts (Masukume et al. 2024). While the Republic of Ireland enacted the recommended public health mitigation measures against the COVID‐19 pandemic, the summative and sustained stress effect on the maternal–fetal dyads might not have been equivalent to that in other countries, with the counterbalancing supportive initiatives adopted by the authorities in the Republic (Philip et al. 2020).

Later, in December 2020, 9 months after the pandemic's onset, the SRB in Northern Ireland rose significantly to 54.48% (119.68 males per 100 females) marking a distinct peak in the study period. This December peak, observed also in England and Wales, could be indicative of a higher frequency of sexual intercourse among partners living together in the United Kingdom, following the March 2020 lockdown (Guerrero 1974; Masukume et al. 2023; Wignall et al. 2021). This is because higher levels of sexual activity can lead to more conceptions outside the most fertile period, which potentially biases toward male births (James 2003). In contrast, while Northern Ireland experienced a December peak in SRB, the Republic of Ireland did not exhibit the same pattern, instead seeing a sharp decline in December, suggesting potentially reduced sexual activity across the entire population during the same period, in alignment with the country's public health guidelines at the start of the pandemic, including a formal recommendation to reduce sexual intercourse during the lockdown period (Masukume et al. 2024).

In Northern Ireland, as in England and Wales, excess mortality rates during the COVID‐19 pandemic were similarly high, particularly during 2020 and 2021, when the impact of COVID‐19 and related factors (such as healthcare strain and lockdown disruptions) led to a significant number of deaths above expected levels (COVID‐19 Excess Mortality Collaborators. 2022). This may explain why both regions saw a notable decline in SRB 3–5 months after the pandemic onset, as COVID‐19 was a high source of stress in both regions. In contrast, the Republic of Ireland experienced one of the lowest excess mortality rates globally during the same period, which may explain the absence of the same “stress pattern” in SRB observed 3–5 months later.

The lack of consistency in time periods affected across regions of the United Kingdom suggests that stress induced by COVID‐19 impacted male fetuses in different gestational periods. In Northern Ireland, England and Wales, the decline in SRB at 5 and 3 months, respectively, suggests that male fetuses were most adversely affected during the fourth (9 minus 5) and sixth month (9 minus 3) of pregnancy, the second trimester of pregnancy. In contrast, in the Republic of Ireland, the dip in the SRB at 9 months instead suggests a conception‐related selection effect, where males were less likely to be conceived. Both mechanisms are possible under the Trivers–Willard hypothesis, which proposes selection against males during times of population‐level stress, and the differences in timing across regions suggest that these effects may vary by local context. However, these patterns are also influenced by the level of sexual activity in the population, making it difficult to disentangle these mechanisms. Specifically, less frequent sexual activity leads to the bulk of conceptions occurring mainly around the midpoint of the fertile period, which biases towards female births and reduces the SRB (James 2003). Northern Ireland's SRB trends align closely with those in England and Wales, pointing toward a shared demographic response across the United Kingdom. This suggests that the SRB shifts in Northern Ireland were likely influenced by pandemic policies, socio‐political contexts, and public health measures similar to those in England and Wales. In contrast, the Republic of Ireland, while sharing the same island (Royle 2012), had a distinct response to the pandemic, likely due to differences in public health policies and socio‐political contexts, leading to different SRB trends (Ahmed and May 2021). One example of this alignment is that Northern Ireland's response, like that of England and Wales, closely followed the timeline of the United Kingdom, with restrictions introduced around 20 March, whereas the Republic of Ireland implemented lockdown measures earlier, on 12 March. This timing difference illustrates how Northern Ireland's approach mirrored that of the United Kingdom, rather than the Republic of Ireland (Colfer 2020).

In August 2020, 5 months after the COVID‐19 pandemic was declared in March, the SRB in Northern Ireland dropped significantly to 49.13%. This decline is strikingly similar to changes observed after other major traumatic events, such as the 2011 Norway terrorist attacks and the 2012 Sandy Hook Elementary School shooting, where the SRB likewise fell to around 49% within 3–5 months (Masukume et al. 2017). Similarly, in South Africa, the SRB decreased 3–5 months after the pandemic was declared (Masukume et al. 2022). The observed reduction strongly suggests a link between the pandemic and increased male fetal loss in Northern Ireland. Furthermore, these results support the Trivers–Willard hypothesis, which suggests that during times of crisis, when environmental conditions are harsh, the birth of males may decrease in favor of females, as females are more likely to survive and reproduce under such conditions (Trivers and Willard 1973).

The media played a pivotal role in disseminating COVID‐19–related information, which surged dramatically worldwide in March 2020, shaping public behaviors and responses (Ng et al. 2021). In the Republic of Ireland, the Taoiseach's (Prime Minister's) initial addresses in March, delivered with empathy and authority, likely reflected his medical background, fostering public trust and a sense of calm (Rafter 2022). In contrast, messaging in the United Kingdom, including Northern Ireland, often framed the pandemic as a “war against COVID‐19,” emphasizing resilience over reassurance (McVittie 2023). These differing communication strategies may have contributed to variations in stress response and subsequent SRB patterns, with Northern Ireland aligning more closely with England and Wales due to shared United Kingdom‐wide messaging.

Stillbirth rates can provide additional support for the Trivers–Willard hypothesis if fewer male live births occurred during 2020 than 2019. However, the singleton stillbirth rate in Northern Ireland, excluding terminations of pregnancy and births prior to 24 + 0 weeks' gestation, was similar in both years: 3.28 per 1000 total births in 2019 and 3.14 per 1000 total births in 2020 (Draper et al. 2022). Notably, the decline in the SRB was observed 5 months after the March 2020 pandemic declaration, suggesting a disproportionate loss of male fetuses. These losses likely occurred among pregnancies at approximately 16 weeks' gestation in March 2020 (i.e., 9 months minus 5 months). As such, they may not be captured in official stillbirth statistics, which are restricted to births at or beyond 24 + 0 weeks' gestation.

The demographic profile of women giving birth in 2020 closely mirrored that of 2019, suggesting minimal change in maternal characteristics. For example, the percentage of mothers aged 35 years and older remained almost identical—24.3% in 2019 compared to 24.5% in 2020 (Health and Social Care Northern Ireland 2023). This consistency indicates that shifts in the composition of individuals choosing to conceive are unlikely to explain the observed findings. In early 2020, certain hospitals in the Republic of Ireland reported an unexpected decline in preterm births and very low birth weight (VLBW) infants (McDonnell et al. 2020; Philip et al. 2020) a trend that was also observed in some other countries (Calvert et al. 2023). Future research should explore the SRB within this vulnerable subgroup, particularly whether male infants were disproportionately represented. Unfortunately, such data were not available for analysis in the present study.

The high energetic demands and vulnerability of twin pregnancies may influence birth patterns under stress, as seen in Norway (Catalano et al. 2021) and the United States (Bruckner et al. 2023), where male twin births declined after COVID‐19's onset. In Northern Ireland, however, the lack of publicly available data on twin births by sex prevented similar analysis.

Although the SRBs for July and October 2020 also fell outside the 95% prediction intervals, we interpreted them cautiously. July, while within the 3–5 months poststressor window, deviated in the opposite direction from expected. October showed a positive deviation, though it was less pronounced than the December peak and fell outside the most established windows of interest. Given the relatively small number of live births in Northern Ireland, raising the likelihood of false positives due to increased month‐to‐month variability (Button et al. 2013), our interpretation focused on August and December, which showed the most extreme deviations for the entire study period and aligned with established theory both in timing and direction (Fukuda et al. 1998; Masukume et al. 2017). Nonetheless, as understanding of these patterns evolves, future research may consider the deviations observed in July and October. Robust research needs many lines of evidence, and interpreting such deviations benefits from considering not just statistical significance, but also theoretical coherence and contextual plausibility (Munafò and Davey Smith 2018).

## 5 Strengths and Limitations

The ecological approach used in this study provides a broad population‐level perspective on SRB trends during the pandemic, although individual‐level data cannot be inferred from these findings due to the potential for ecological fallacy (Björk et al. 2021; Pearce 2011). Additionally, this study did not investigate SRB fluctuations in nonpandemic years, such as the peaks and dips recorded in 2019 and 2017, as these variations were not hypothesized a priori. A key strength of this study is the use of live birth data sourced from a complete civil registration and vital statistics system, ensuring that missing data is not a concern (Adair et al. 2023). Additionally, the integration of data on policy differences and media messaging among regions, along with demographic profiles over time, strengthened our ability to interpret the findings.

## 6 Conclusions

This study provides evidence that the COVID‐19 pandemic and the public health mitigation measures adopted had a significant impact on the SRB in Northern Ireland, likely due to the increased population stress that disproportionately affected male fetuses. The SRB decline in August 2020, observed 3–5 months after the onset of the pandemic, aligns with patterns seen in other regions experiencing similar population‐wide stressors. Additionally, the December 2020 SRB peak, despite a decline in the overall birth rate, suggests that lockdown measures led to increased sexual activity within a subset of the population, resulting in higher conception rates in that same subset. The SRB trends observed in Northern Ireland align more closely with those in England and Wales than with the Republic of Ireland, indicating that socio‐political factors within the United Kingdom may have played a more significant role than geographical proximity in influencing demographic responses to the pandemic.

These findings highlight the potential of SRB as a valuable indicator for tracking population health during public health crises and highlight the importance of SRB monitoring in future pandemic preparedness efforts.

## Ethics Statement

The authors have nothing to report.

## Conflicts of Interest

The authors declare no conflicts of interest.

## Supporting information


**Supplementary Table 1** Procedure for selecting parameters of the autoregressive moving average (ARMA) model used to estimate and predict the sex ratio at birth in Northern Ireland.

## Data Availability

The data that support the findings of this study are available from the Northern Ireland Statistics and Research Agency *Gníomhaireacht Thuaisceart Éireann um Staitisticí agus Taighde* https://www.nisra.gov.uk/.
