# Supplementary material for: Sex Ratio at Birth in Northern Ireland During the COVID‐19 Pandemic: A Comparison With Published Data From the Republic of Ireland, England and Wales
Source: Am J Hum Biol. 2025 Jun 27;37(7):e70099. doi: 10.1002/ajhb.70099 (PMC12205282; doi:10.1002/ajhb.70099)
Supplement: Supplementary file 1 — Supplementary Table 1 Procedure for selecting parameters of the autoregressive moving average (ARMA) model used to estimate and predict the sex ratio at birth in Northern Ireland. [file AJHB-37-e70099-s001.docx]

**Appendix**

**Supplementary Table 1.** Procedure for selecting parameters of the autoregressive moving average (ARMA) model used to estimate and predict the sex ratio at birth in Northern Ireland.

| AR | MA | AR | | | | |  | MA | | | | | AIC |
| --- | --- | --- | --- | --- | --- | --- | --- | --- | --- | --- | --- | --- | --- |
|  |  | L1 | L2 | L3 | L4 | L5 |  | L1 | L2 | L3 | L4 | L5 |  |
| 0 | 0 |  |  |  |  |  |  |  |  |  |  |  | -356.93 |
| 0 | 1 |  |  |  |  |  |  | -0.05 |  |  |  |  | -355.06 |
| 0 | 2 |  |  |  |  |  |  | -0.00 | -0.15 |  |  |  | -353.52 |
| 0 | 3 |  |  |  |  |  |  | 0.10 | -0.14 | 0.19 |  |  | -353.14 |
| 0 | 4 |  |  |  |  |  |  | 0.11 | - -0.08 | 0.33 | -0.47 |  | -361.41 |
| 0 | 5 |  |  |  |  |  |  | 0.10 | -0.08 | 0.33 | -0.47 | 0.02 | -359.44 |
| 1 | 0 | -0.04 |  |  |  |  |  |  |  |  |  |  | -355.05 |
| 1 | 1 | 0.92 |  |  |  |  |  | -1.00 |  |  |  |  | -354.48 |
| 1 | 2 | -0.70 |  |  |  |  |  | 0.81 | -0.19 |  |  |  | -357.43 |
| 1 | 3 | -0.56 |  |  |  |  |  | 0.73 | 0.04 | 0.31 |  |  | -356.73 |
| 1 | 4 | -0.03 |  |  |  |  |  | 0.14 | -0.08 | 0.33 | -0.46 |  | -359.43 |
| 1 | 5 | 0.72 |  |  |  |  |  | -0.72 | -0.12 | 0.41 | -0.68 | 0.52 | -360.89 |
| 2 | 0 | -0.05 | -0.06 |  |  |  |  |  |  |  |  |  | -353.30 |
| 2 | 1 | -0.91 | -0.18 |  |  |  |  | 1.00 |  |  |  |  | -357.84 |
| 2 | 2 | -1.14 | -0.36 |  |  |  |  | 1.23 | 0.23 |  |  |  | -356.12 |
| 2 | 3 | -0.77 | -0.27 |  |  |  |  | 0.93 | 0.37 | 0.44 |  |  | -357.96 |
| 2 | 4 | -0.08 | -0.09 |  |  |  |  | 0.18 | -0.01 | 0.35 | -0.47 |  | -357.55 |
| 2 | 5 | 1.04 | -0.35 |  |  |  |  | -1.07 | 0.14 | 0.52 | -0.85 | 0.76 | -361.96 |
| 3 | 0 | -0.04 | -0.06 | 0.16 |  |  |  |  |  |  |  |  | -352.86 |
| 3 | 1 | -0.89 | -0.10 | 0.10 |  |  |  | 1.00 |  |  |  |  | -356.38 |
| 3 | 2 | -0.85 | -0.07 | 0.10 |  |  |  | 0.97 | -0.03 |  |  |  | -356.38 |
| 3 | 3 | NC | NC | NC |  |  |  | NC | NC | NC |  |  | NC |
| 3 | 4 | -0.36 | -0.51 | -0.37 |  |  |  | 0.45 | 0.52 | 0.73 | -0.34 |  | -361.23 |
| 3 | 5 | 1.01 | -0.19 | -0.16 |  |  |  | -1.06 | 0.03 | 0.63 | -0.79 | 0.68 | -362.63 |
| 4 | 0 | 0.02 | -0.08 | 0.16 | -0.35 |  |  |  |  |  |  |  | -358.28 |
| 4 | 1 | 0.06 | -0.08 | 0.16 | -0.36 |  |  | -0.05 |  |  |  |  | -356.31 |
| 4 | 2 | 0.19 | -0.38 | 0.15 | -0.39 |  |  | -0.22 | 0.36 |  |  |  | -355.18 |
| 4 | 3 | 0.29 | 0.12 | -0.58 | -0.35 |  |  | -0.30 | -0.30 | 1.00 |  |  | -358.36 |
| 4 | 4 | 0.30 | 0.11 | -0.58 | -0.33 |  |  | -0.32 | -0.30 | 1.01 | -0.02 |  | -360.36 |
| 4 | 5 | 1.01 | -0.19 | -0.15 | -0.01 |  |  | -1.05 | 0.03 | 0.62 | -0.78 | 0.68 | -358.63 |
| 5 | 0 | 0.01 | -0.08 | 0.16 | -0.35 | -0.03 |  |  |  |  |  |  | -356.32 |
| 5 | 1 | -0.17 | -0.07 | 0.14 | -0.32 | -0.10 |  | 0.18 |  |  |  |  | -354.35 |
| 5 | 2 | 1.22 | -0.60 | 0.24 | -0.56 | 0.42 |  | -1.30 | 0.64 |  |  |  | -356.41 |
| 5 | 3 | 0.98 | -0.40 | -0.03 | -0.54 | 0.36 |  | -1.12 | 0.45 | 0.37 |  |  | -358.12 |
| 5 | 4 | 1.15 | -0.37 | 0.11 | -0.62 | 0.41 |  | -1.32 | 0.41 | 0.21 | 0.12 |  | -356.28 |
| 5 | 5 | 0.87 | -0.51 | -0.07 | 0.14 | -0.34 |  | -0.92 | 0.42 | 0.42 | -0.92 | 1.00 | -362.74 |

AIC: Akaike Information Criterion; AR: autoregressive parameter; MA: moving average parameter; NC: not converged.
